# Leaf Extract of *Perilla frutescens* (L.) Britt Promotes Adipocyte Browning via the p38 MAPK Pathway and PI3K-AKT Pathway

**DOI:** 10.3390/nu15061487

**Published:** 2023-03-20

**Authors:** Fancheng Chen, Silin Wu, Dejian Li, Jian Dong, Xiaowei Huang

**Affiliations:** 1 Department of Orthopedic Surgery, Zhongshan Hospital, Fudan University, Shanghai 200032, China; chenfancheng@126.com; 2 Department of Orthopaedics & Rehabilitation, School of Medicine, Yale University, New Haven, CT 06510, USA; 3 Department of Neurosurgery, McGovern School of Medicine, University of Texas Health Science Center, Houston, TX 77030, USA; silin.wu@uth.tmc.edu; 4 Department of Orthopedics, Shanghai Pudong Hospital, Fudan University Pudong Medical Center, Shanghai 200120, China; lidejian880820@163.com; 5 Facutly of Medicine, Eberhard Karls University of Tübingen, 72076 Tübingen, Germany; 6 Department of Orthopaedics, The First Affiliated Hospital of Soochow University, Suzhou 215006, China

**Keywords:** *Perilla frutescens* (L.) Britt, network pharmacology, adipocyte browning, in vitro validation, molecular mechanism

## Abstract

The leaf of *Perilla frutescens* (L.) Britt (PF) has been reported to negatively affect adipocyte formation, inhibit body-fat formation, and lower body weight. However, its effect on adipocyte browning remains unknown. Thus, the mechanism of PF in promoting adipocyte browning was investigated. The ingredients of PF were acquired from the online database and filtered with oral bioavailability and drug-likeness criteria. The browning-related target genes were obtained from the Gene Card database. A Venn diagram was employed to obtain the overlapped genes that may play a part in PF promoting adipocyte browning, and an enrichment was analysis conducted based on these overlapped genes. A total of 17 active ingredients of PF were filtered, which may regulate intracellular receptor-signaling pathways, the activation of protein kinase activity, and other pathways through 56 targets. In vitro validation showed that PF promotes mitochondrial biogenesis and upregulates brite adipocyte-related gene expression. The browning effect of PF can be mediated by the p38 MAPK pathway as well as PI3K-AKT pathway. The study revealed that PF could promote adipocyte browning through multitargets and multipathways. An in vitro study validated that the browning effect of PF can be mediated by both the P38 MAPK pathway and the PI3K-AKT pathway.

## 1. Introduction

Obesity is mainly caused by an imbalance of energy intake and consumption, which is characterized by a large number of lipids accumulated in white adipocytes [1]. Long-term excess white fat deposition not only changes the body shape but also increases the risk of diabetes and hyperlipidemia [2]. Interestingly, the beige adipocytes present in white adipose tissue are similar in structure and function to brown adipocytes, with more lipid droplets, and are rich in mitochondria and highly expressed thermogenesis markers of uncoupling protein 1 (UCP1) [3]. The promotion of adipocyte browning, which can increase the amount of beige or brite adipocytes, increases energy expenditure and could be a potential approach to prevent obesity.

Several important molecules have been reported to play an important role in adipogenesis and browning regulation. Sakaguchi et al. [4] revealed that the phosphatase-binding protein Alpha4 (α4) plays an important part in adipogenesis and mitochondrial thermogenesis. It acts through the insulin signaling pathway. The knockout of α4 leads to impaired adipogenesis as well as thermogenesis but increased insulin resistance. Lee et al. [5] reported that regulated development and DNA damage response 1 (REDD1) upregulation can simulate preadipocyte differentiation through atypical IKK-independent NF-κB activation by sequestering IκBα from the NF-κB/IκBα complex. Adenosine monophosphate-activated protein kinase (AMPK) regulates energy balance and the metabolic switch. AMPK stimulates the catabolic pathway of adenosine triphosphate (ATP) production and shuts down the ATP-consuming anabolic pathway, respectively increasing energy production and expenditure [6]. Therefore, it also has been reported as a vital molecule for adipocyte browning.

Traditional Chinese Medicine (TCM) is a rich resource to inspire novel insights into therapeutic approaches. Several herbs from TCM have been investigated for their pharmacological function in increasing calorie expenditure and reducing fat-tissue formation in mice. Cinnamon, the bark of trees of the *Cinnamomum genus*, has been proven to induce browning in the subcutaneous adipocytes of obese mice. Oral administration of cinnamon extract can promote UCP1 expression in the subcutaneous adipose tissue and reduce the body weight of obese mice [7]. The β3-adrenergic receptor (β3-AR) may be involved in cinnamon-extract-induced browning. Wang et al. [8] found that ginger can promote adipose-tissue browning by regulating the SIRT1/AMPK/PCG-1α pathway and upregulating the gene expression of beige adipocyte-selective markers. As a result, the identification of a safe herb that can promote white adipocyte browning is a feasible strategy for treating obesity and related pathophysiological conditions. It is interesting to note that both of the aforementioned herbs that can promote adipocyte browning belong to the Pungent-Warm Exterior-Releasing medicinal family, according to the theory of TCM. The leaf of *Perilla frutescens* (L.) Britt (PF) is also a member of the Pungent-Warm Exterior-Releasing medicinal family. Thomas et al. [9] found that Purple Perilla (*P. frutescens var. acuta*) leaf extract has anti-obesity effects in rodents and can be effective in obesity management due to its ability to reduce lipid accumulation in differentiated adipocytes and prevent an increased body weight in C57BL/6J mice fed high-fat diets. Feng et al. [10] found that PF extract demonstrated a negative effect on adipocyte formation from 3T3L-1 pre-adipocytes. Another study demonstrated that PF extract can inhibit body-fat formation and lower the body weight of obese mice [11]. Consequently, it could be postulated that PF may also promote adipocyte browning.

PF extract comprises more than one component ingredient, indicating complicated pharmacological targets and mechanisms. So far, most existing studies on herb extracts have concentrated on single or limited ingredients and targets, lacking an integral exploration of the pharmacological mechanisms of the herb [12,13]. As a result, it poses a challenge to systemically investigate the reaction ingredients of the PF extract on vital molecules to promote browning. Since the traditional approaches are limited, a novel systemic approach, network pharmacology, which was established based on the theory of Shao Li published in 2013, was employed in the present study [14]. Network pharmacology, as a in silico technique, focuses on the synergy of multicomponent and multitarget systems, which is very suitable for plant extract study. It has emerged as a useful tool for understanding the fine details of drug–target interactions [15]. Network-based topological analysis tools, particularly dynamic analysis tools, have great potential for finding and developing multitarget drugs and identifying alternative targets [16]. Molecular docking is a computational simulation to explore ligand conformations adopted within receptor binding sites during intermolecular recognition [17]. Recently, it has also drawn great attention in the field of plant research [18,19]. Singh et al. [20] used molecular docking, long-term molecular dynamics, and molecular-mechanics Poisson–Boltzmann surface-area analysis to find out the potential of quinoline-based molecules as allosteric inhibitors.

**Figure 1:**
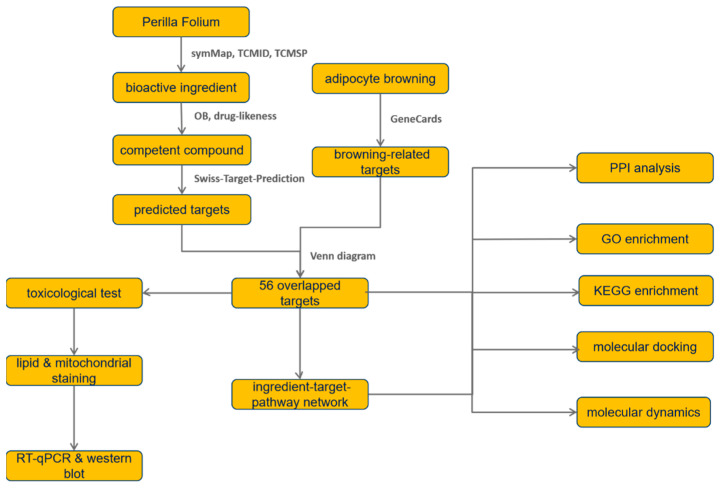
The overall workflow of the study.

In the present study, network pharmacology methods were used in combination with molecular docking to reveal the potential molecular mechanisms of PF promoting adipocyte browning, and results were validated by experimental evidence. The overall workflow is shown in Figure 1.

## 2. Materials and Methods

### 2.1. Leaf of Perilla frutescens (L.) Britt (PF) Bioactive Ingredient Acquisition

The ingredients in PF were obtained through the symMap database, the Traditional Chinese Medicines Integrated Database (TCMID), and the Traditional Chinese Medicine Systems Pharmacology Database and Analysis Platform (TCMSP) [17]. The chemicals with oral bioavailability ≥30% and with violations of no more than two items in the five criteria of drug-likeness were screened out as competent pharmacological compounds for further study. The drug-likeness information of each ingredient can be retrieved from the SwissADME database (http://www. swissadme.ch, accessed on10 August 2022) [21].

### 2.2. Prediction of Protein-Encoding Target Genes Based on Compound Structures

Swiss-Target-Prediction [22], a website database tool, was used for target-protein prediction. The SMILE formula of each compound was retrieved from the PubChem database and then input into the online tool for drug-target prediction. Targets with a prediction probability of less than 0.1 were excluded.

### 2.3. Search for Browning-Related Targets

Adipocyte browning-related genes were found in the GeneCards database; “adipocyte browning” and “adipose browning” were used as search keywords. Protein-encoding genes with a disease relevance score ≥3 were chosen for further investigation.

### 2.4. Topological Analysis for the Interactions among Overlapped Target Genes

The overlapped genes of both PF-related genes and browning-associated targets were filtered by a Venn diagram. Based on the aforementioned overlapped genes, a topological interaction network was created after inputting the list of genes into the String database, intending to screen out the hub genes. For the topological network establishment, the interaction score was set as 0.4. Further topological analysis was performed using the MCODE plugin of Cytoscape, which was used to screen out the core clusters from the entire network.

### 2.5. Enrichment Analysis Based on GO and KEGG Database

The potential pathways and biological functions of the overlapped genes were predicted using enrichment analysis. The R package “cluster profile” was used to execute the analysis. The enriched items with an adjusted *p*-value < 0.01 were set as the cutoff values.

### 2.6. Exploration of the Interactions among Ingredients, Targets, and Pathways

To further elucidate the correspondence between the active ingredient and overlapped target proteins, the ingredient–target network was constructed. Furthermore, to study the connection between protein targets and the 15 top pathways, the targets–pathway correspondence was also added, and the ingredient–target–pathway (I-T-P) network was established. To characterize the role of active ingredients in the network, three topological parameters were measured, namely degree, betweenness, and closeness [16].

### 2.7. Molecular Docking Simulation

The top seven active ingredients filtered from the I-T-P network and the top seven targets screened based on the PPI network were paired for a molecular docking simulation, which was conducted using the AutoDock software package [23]. For the preprocessing stage, the 2D structures of ingredients were acquired from the PubChem database, which were then transformed into 3D structures as well as optimized with minimal energy using Chem3D software and converted to the pdbqt format. The protein crystal structure was obtained for the PDB protein bank. The 3D structures of AKT1 (PDB id: 1UNP), CASP3 (PDB id: 1NME), EGFR (PDB id: 1M14), MAPK14 (PDB id: 1A9U), PPARG (PDB id: 1I7I), SRC (PDB id:1O41), STAT3 (PDB id: 6NJS), and VEGFA (PDB id: 1MJV) were then imported and preprocessed in the PyMOL software for dehydrated, hydrogenated, and pdbqt format conversion. The final calculations of the docking and binding energies were conducted using AutoDock Vina 4.2. The docking results were visualized in the Pymol software. The estimated binding affinity score ΔG (kcal/mol) was calculated to characterize the compound- and protein-binding force [24]. A lower binding-affinity score indicated a better binding affinity [25]. The results were visualized by Discovery Studio 2012 and visualized using a heatmap plot.

### 2.8. Molecule Dynamics of Ligand and Target Combination

The binding of docked ligand–receptor complex systems was further validated by molecular dynamics simulations using the AMBER 18 software [26]. For the preprocessing stage, Gaussian 09 software [27] was employed to set the charges of the ligand. The GAFF2 small-molecule force field and the ff14SB protein force field [28] were assigned to ligand and protein receptors, respectively. The LEaP module was used to add hydrogen atoms to the system, a truncated octahedral TIP3P solvent box was added at a distance of 10 Å from the system, Na^+^/Cl^−^ was added to the system to balance the system charge, and finally, the topology and parameter files for the simulation were output. The system first underwent energy optimization and was then heated to 298.15 K. A 500 ps NVT (isothermal) phylogenetic simulation was then conducted and an equilibrium simulation of 500 ps was performed. At the final stage, 100 ns NPT (isothermal isobaric) phylogenetic simulations were performed. The geometrical parameters of the systems, such as the root mean square deviation (RMSD) and root mean square fluctuation (RMSF), were determined and compared with the primitive ligand complex system.

### 2.9. Preparation of PF Extract

The *Perilla frutescens* (L.) Britt leaves were purchased in the local market, and then dried and crushed into powder. Dried PF powder was dissolved in 70% ethanol in a 1:30 (g/mL) ratio [29,30]. The solution was kept in a water bath at 60 °C for 1 h and then centrifuged at 3000 r/min for 10 min. The supernatant was filtrated, and the recovered ethanol fraction was concentrated. The extraction procedure was repeated three times, combining the three extracts. The mixed ethanol supernatant of all extracts was then filtered, concentrated, freeze-dried into a powder, and stored at 4 °C for later use [29,31].

### 2.10. Cell Culture and Differentiation

Primary mouse-bone-marrow mesenchymal stem cells (BMSCs) [32] were cultured in Dulbecco’s modified Eagle’s medium (DMEM; Shenggong, Shanghai, China) supplemented with 10% bovine calf serum and 1% penicillin–streptomycin (Biyuntian, Wuhan, China). When the cell density reached 80% confluence, cells were seeded. Two days after confluence, the cells were cultured with an adipogenic differentiation medium containing 0.5 mM isobutylmethylxanthine, 1 μM dexamethasone, 10 μM rosiglitazone, and 10 μg/mL insulin (ADP1, Guangzhou Bojin Biotechnology Co., Ltd., Guangzhou, China) supplemented with 10% fetal bovine serum and 1% penicillin–streptomycin (Biyuntian, Wuhan, China) to induce adipocyte differentiation. The time point was set as Day 0.

After 2 days, the ADP1 medium was changed to a maintenance medium (ADP2, Guangzhou Bojin Biotechnology Co., Ltd.), including 10 μg/mL insulin only, until Day 10. PF was added to the BMSCs starting from Day 0 and the medium changed every three days. For the inhibitor test, BMSCs were pretreated with AKT inhibitor SC394003 (10 μM, Santa Cruz, CA, USA) and p38 inhibitor (SB203580) (10 μM, Sigma-Aldrich, Munich, Germany) for 24 h and then simulated with the same fresh medium for 30 min before sample collection.

### 2.11. Cell Viability Assay

The Cell Counting Kit-8 (CCK8, Dojindo, Kumamoto, Japan) assay was employed to determine cell viability. BMSCs were seeded and incubated with PF (0, 50, 100, 200, 400, 800, and 1600 μg/mL). After 10 days of induction, the cells were washed with PBS, and 10% CCK-8 solution diluted with alpha-MEM medium was added to each well; then, they were incubated for an hour, and the absorbance at 450 nm was measured by a microplate reader (Thermo, Waltham, MA, USA).

### 2.12. Real-Time Quantitative Polymerase Chain Reaction (RT-qPCR)

Real-time quantitative polymerase chain reaction (RT-qPCR) [33] was used to characterize adipogenesis-related gene expression. The total RNA of the BMSCs was extracted using TRIzol reagent and then reversely transcribed to complementary DNA (cDNA). RT-qPCR was carried out with SYBR Green Master Mix, following the manufacturer’s instructions, using an ABI PRISM 7500 PCR Sequence Detection System (Applied Biosystems, Foster City, CA, USA), and the melting curve was tested simultaneously. The primers are listed in Supplemental Table S1.

### 2.13. Western Blot Analysis

Cells were lysed to obtain the total protein content using freshly prepared radioimmunoprecipitation assay buffer (RIPA) at 4 °C [33]. Protein was then normalized and separated by 10% sodium dodecyl sulfate-polyacrylamide gel (SDS PAGE) and transferred to a PVDF membrane (Millipore, Burlington, MA, USA). The membrane was subsequently blocked and incubated with a diluted primary antibody. Finally, it was incubated with a secondary antibody for visualization using an electrochemiluminescence detection reagent (SAB, College Park, MD, USA). The grayscale bands were analyzed by ImageJ (National Institutes of Health, Bethesda, MD, USA) software. Actin was used as an internal control. Actin, PI3K, P-PI3K, AKT, P-AKT, STAT3, P-STAT3, and PPARG antibodies with a dilution ratio of 1:3000 were purchased from Servicebio (Wuhan, China). UCP1, CREB, and P-P38 antibodies with a dilution ratio of 1:1000 were purchased from Servicebio Co. (Wuhan, China). P38 and P-CREB antibodies with a dilution ratio of 1:1000 were purchased from Abcam (Cambridge, UK)

### 2.14. Determination of Lipid Accumulation

The Oil Red O (ORO) staining method [34] was employed to characterize the intracellular lipid accumulation, according to the manufacturer’s instructions. After incubation for 30 min, the stained lipid droplets were viewed under a microscope. The lipid accumulation was quantified by solving in isopropanol, and then the absorbance at 520 nm was checked.

### 2.15. Mitochondrial Mass Measurement

The BMSCs that were treated with PF or left untreated for 10 days were washed with PBS and incubated with DMEM containing 100 nM MitoTracker Green FM (Beyotime Biotechnology, Jiangsu, China) for 30 min at 37 °C [35]. Cells were washed with PBS and then incubated in a prewarmed DMEM medium at 37 °C. Fluorescent intensity at 490 nm was observed using an Olympus confocal microscope (FV10-MCPSU).

### 2.16. Statistical Analysis

Statistical analysis was conducted using Prism 8 software. Data were expressed as the mean ± SD and analyzed using one-way ANOVA. Differences between groups were considered to be statistically significant if values of *p* < 0.05.

## 3. Results

### 3.1. Putative Targets of PF Activating Browning

**Figure 2:**
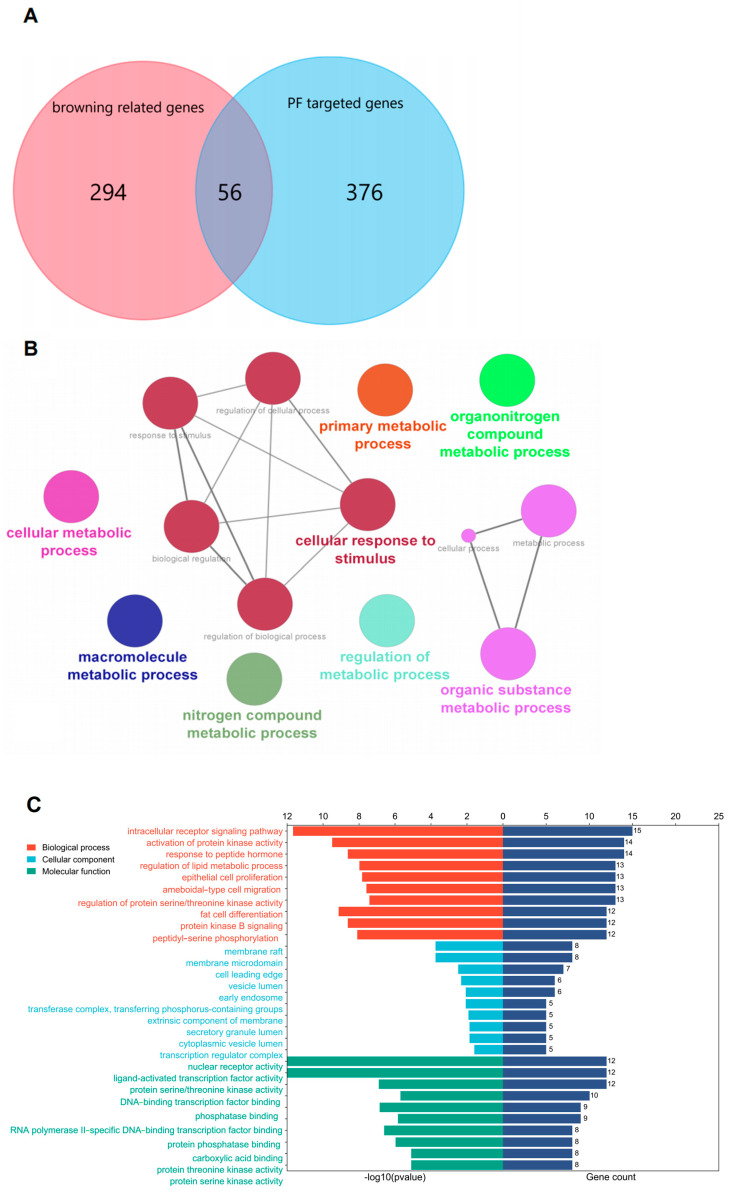
(**A**). Venn diagram of 56 overlapped targets between browning-related genes and PF-predicted genes. (**B**). Biological process of 56 overlapped genes analyzed by Glue GO plugin. (**C**). GO enrichment analysis of 56 overlapped genes using the cluster profile package of R language.

There were 396 compounds in PF obtained from those the aforementioned databases. After screening by oral bioavailability and drug-likeness criteria, 49 compounds were found. A total of 19 compounds were excluded, as no target could be predicted with a prediction probability of more than 0.1. Overall, 427 PF-predicted genes were finally generated from 30 compounds, and 1493 browning-related genes were found using the GeneCards database, based on which 342 genes were selected with a relevance score of more than 3. Eventually, 56 overlapped genes predicted by 17 compounds from both the PF-predicted genes and browning-related genes were selected using Venn diagrams, as shown in Figure 2A.

### 3.2. Enrichment Analysis

**Figure 3:**
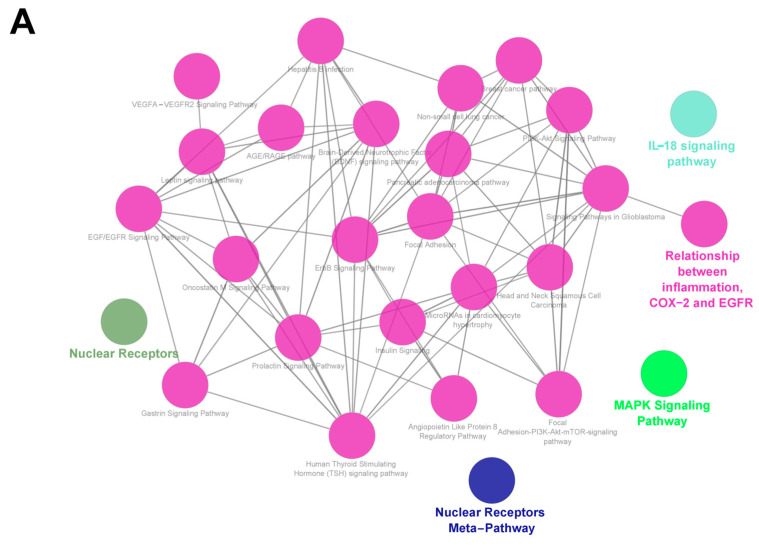

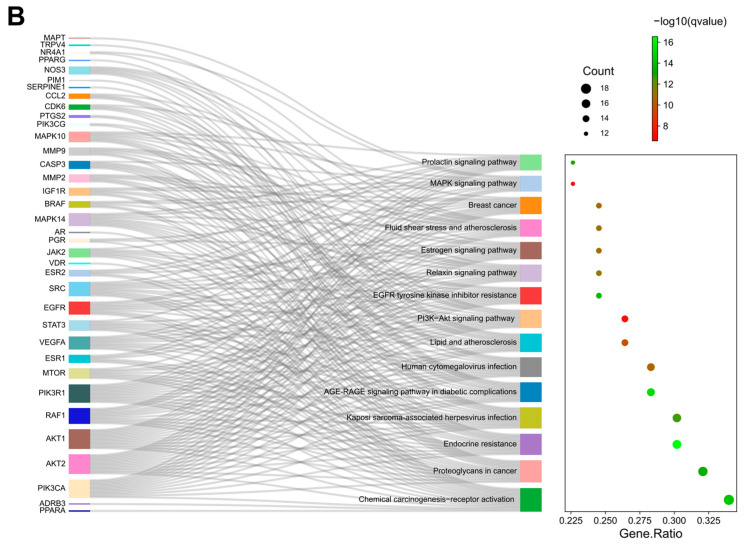
(**A**). Reactome pathway enrichment analysis of 56 overlapped genes. (**B**). KEGG enrichment of 56 overlapped genes.

The enrichment analysis was performed based on the 56 overlapped targets to predict the potential mechanisms of PF promoting adipocyte browning. Overall, 1735 biological process (BPs), 27 (cell components) CCs, and 82 molecular functions (MFs) were enriched in the GO analysis. The biological process enrichment conducted by the Glue GO plugin is shown in Figure 2B, and the top 10 terms in each subitem are illustrated in Figure 2C. It was predicted that PF activating adipocyte browning might occur through the intracellular receptor-signaling pathway, activation of protein kinase activity, response to peptide hormone, and regulation of the lipid metabolic process. In the meantime, target proteins were located in the membrane raft, membrane microdomain, cell-leading edge, vesicle lumen, and early endosome. The reactome pathway analysis indicated that the MAPK signaling pathway, nuclear-receptors metapathway, and IL-18 signaling pathway may be related to the PF-activated browning mechanism, as shown in Figure 3A. The KEGG pathway analysis screened out 155 pathways with statistical significance, including the PI3K-Akt signaling pathway, MAPK signaling pathway, AGE-RAGE signaling pathway in diabetic complications, estrogen signaling pathway, and lipid and atherosclerosis. The top 15 KEGG pathways with the highest gene ratio were selected for visualization, as shown in Figure 3B.

### 3.3. Protein–Protein Interaction (PPI) Network of Targets for ZYS Promoting Adipocyte Browning

**Figure 4:**
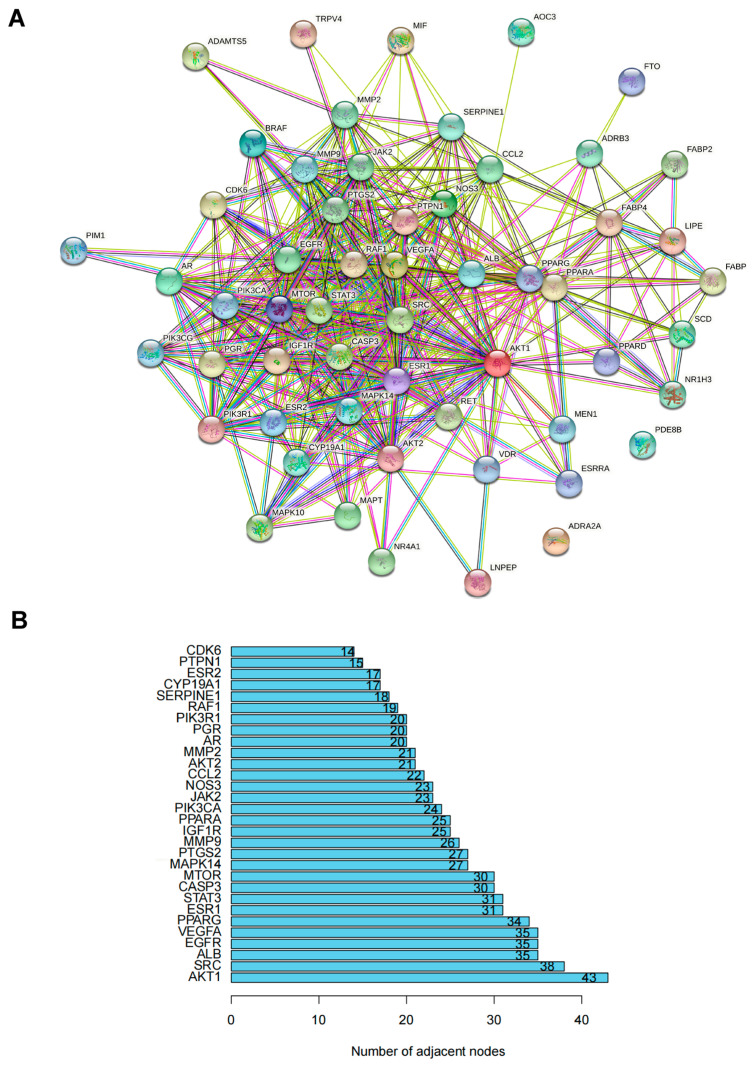

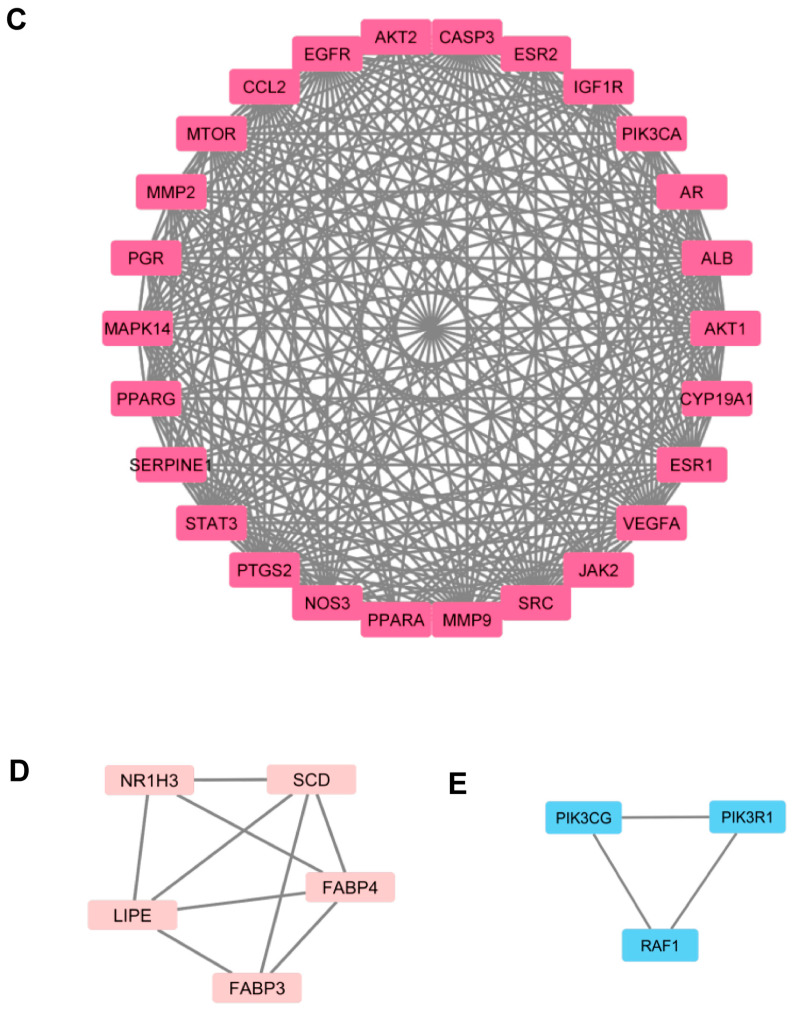
(**A**). PPI analysis of 56 overlapped genes. 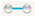 indicates known interactions from curated databases. 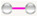 indicates known interactions experimentally determined. 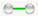 represents predicted interactions with gene neighborhood. 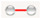 represents predicted interactions with gene fusions. 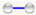 represents predicted interactions with gene co-occurrence. 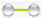 indicates interaction from textmining. 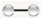 indicates interaction from co-expression. 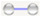 indicates interaction from protein homology. (**B**). Ranking of the top 30 nodes from the PPI network according to the number of adjacent nodes. (**C**). Cluster 1 filtered by MCODE plugin. (**D**). Cluster 2 filtered by MCODE plugin. (**E**). Cluster 3 filtered by MCODE plugin.

PPI analysis, as plotted in Figure 4A, was conducted on 56 common genes. The top 30 proteins with the most adjacent nodes are plotted in Figure 4B. These proteins may play a significant part in activating adipocyte browning. The core cluster genes were filtered by the MCODE plugin, as shown in Figure 4C–E. It also seems that the function of cluster 1 plays a major role in promoting adipocyte browning, as it includes most of the genes.

### 3.4. Ingredient–Target–Pathway (I-T-P) Network Construction

**Figure 5:**
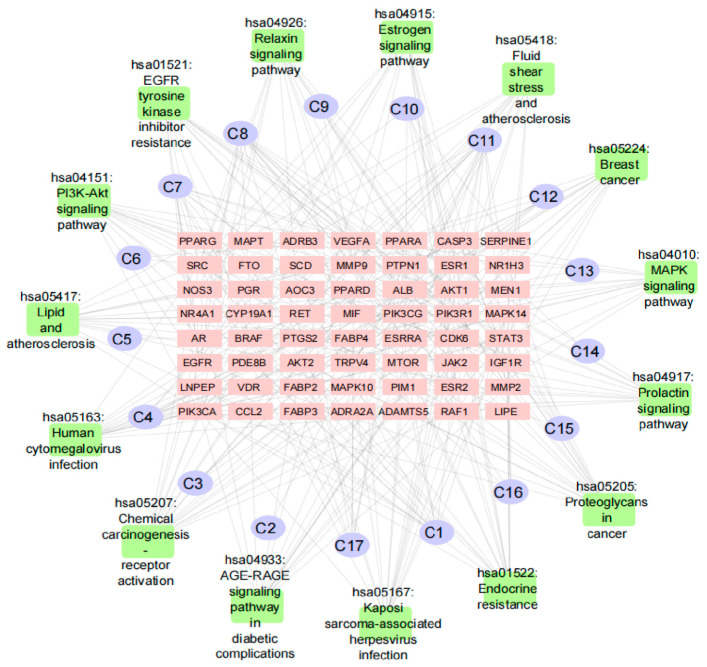
Network of 17 druggable compounds and 56 target genes related to browning. Purple ellipses represent druggable compounds. Pink rectangles are overlapped target genes. Green rectangles are the top 15 KEGG pathways.

The I-T-P network was established to illustrate the ingredients and their correspondence to the receptors and pathway involved, including 88 nodes and 312 edges, as shown in Figure 5.

The top five compounds with the highest edge numbers were luteolin (C8), nerolidyl acetate (C11), 1-(2,4,5-triethoxyphenyl) propane-2-amine (C1), isoeugenol (C7), and dibutyl phthalate (C4). The mean topological parameters of the top five compounds were 12 degrees, 0.0728 of node betweenness, and 0.3506 of closeness. The top five target nodes with the most connections were AR, ESR1, PTGS2, PTPN1, and SRC. The mean value of topological parameters was 8 degrees, 0.0398 of node betweenness, and 0.3442 of closeness, respectively. The 17 compounds of PF are listed in Table 1.

### 3.5. Molecular Docking Demonstrating Compound–Protein Interaction

**Figure 6:**
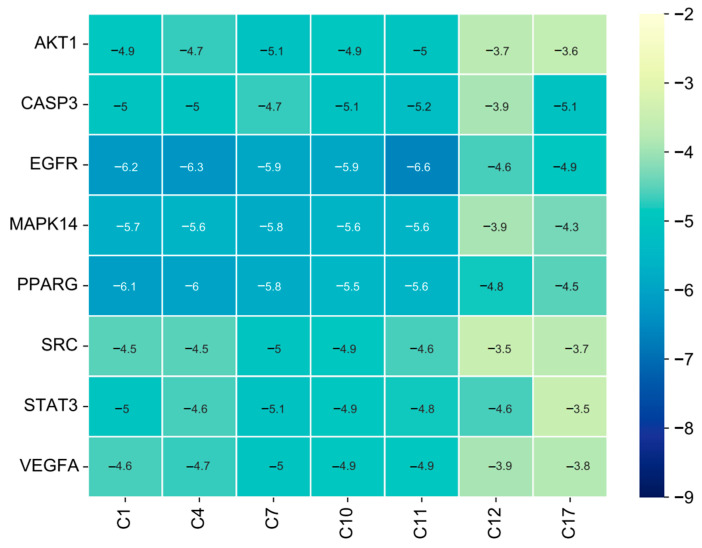
Heat map of binding energy (kcal/mol) between top eight key targets and seven core ingredients.

To further elucidate the combination of compound and predicted proteins, a molecular docking simulation was performed. As the lowest binding energy indicated the most stable binding modality, the binding pattern with the lowest binding energy was simulated. Based on the PPI and enrichment results, eight key browning-related targets (AKT1, CASP3, EGFR, MAPK14, PPARG, SRC, STAT3, and VEGFA) and seven compounds with top-degree values in the I-T-P network were paired for molecular docking. The binding energy results are summarized in the heat map shown in Figure 6.

**Figure 7:**
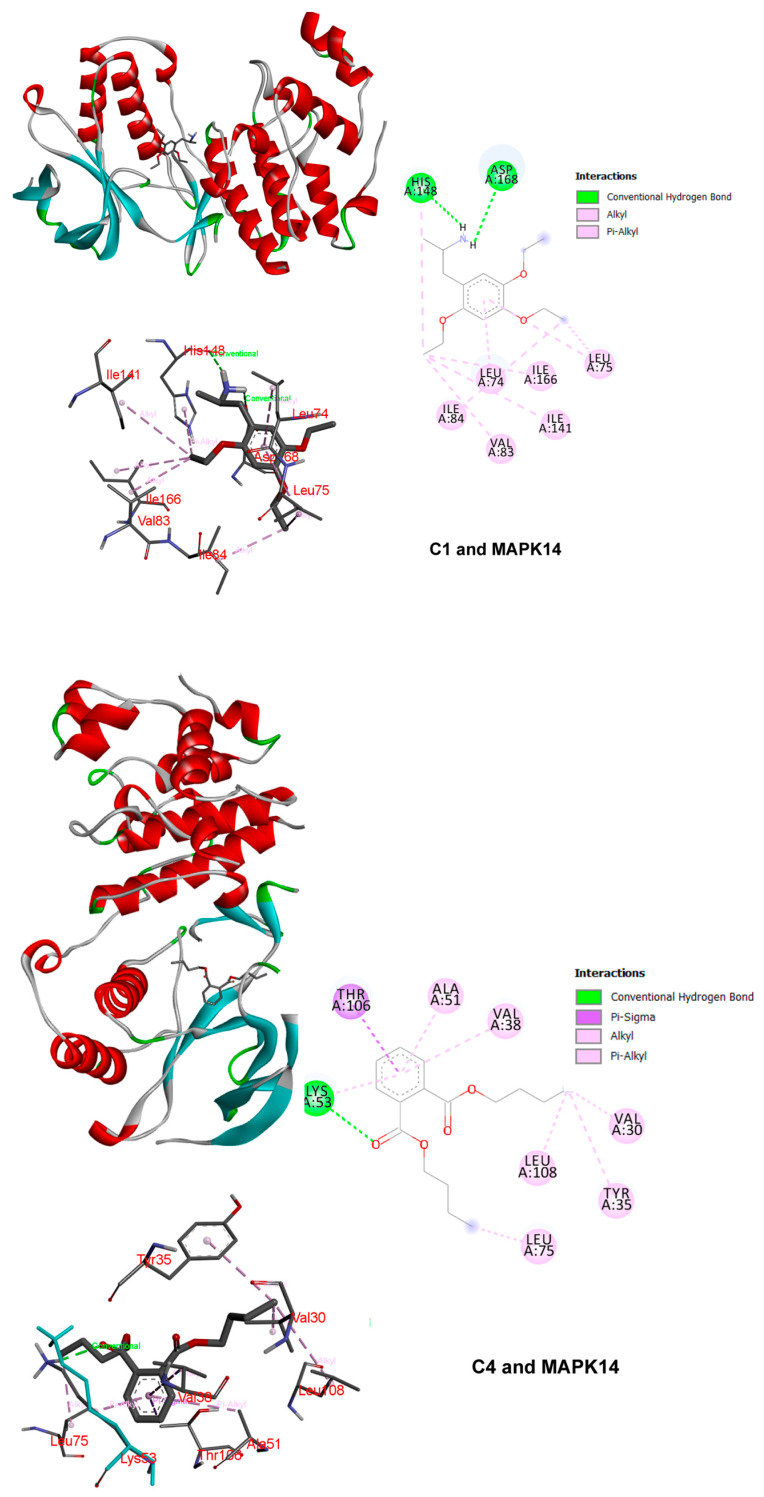

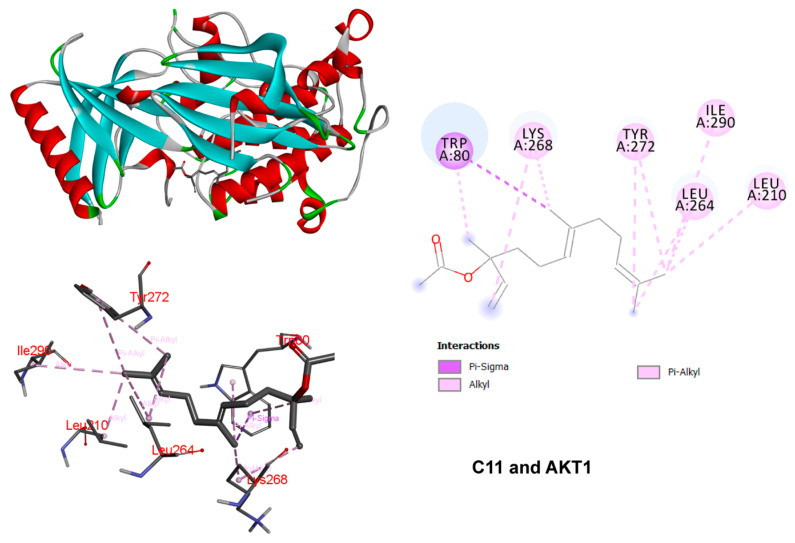
The 3D and 2D binding patterns of MAPK14 and AKT1 with ligands. The left part is the overall binding pattern of protein and ligands. The middle part represents the 3D binding pattern of ligands with surrounding amino acid residues. The right part is the 2D illustration of the binding pattern of ligands with surrounding amino acid residues. Different colors represent different binding patterns.

It is generally accepted that a binding energy less than −4.25 kcal/mol indicates specific binding activity of the ligand to the receptor, while a binding energy less than −5.0 kcal/mol indicates better binding activity [36]. According to the affinity score, MAPK14 can bind with C1 and C4; the binding mode included van der Waals, pi-alkyl, alkyl, pi-sigma, and conventional hydrogen bonds. AKT1 can also bind with C11; the binding mode included pi-alkyl, alkyl, and pi-sigma. The binding patterns and the connected amino acid residues are plotted in Figure 7.

### 3.6. Molecular Dynamics Simulation of Ligand Complex

**Figure 8:**
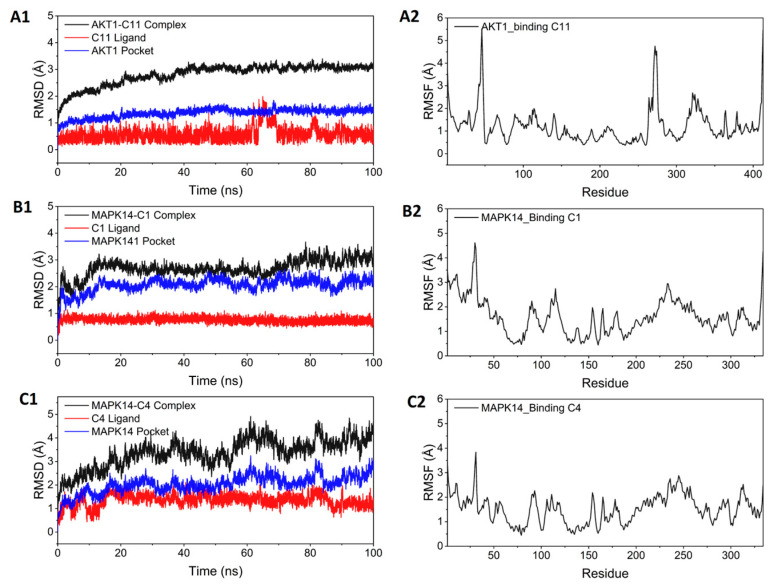
(**A1**–**C1**). Variation in complex root mean square deviation (RMSD) difference over time during molecular dynamics simulations. (**A2**–**C2**). Root mean square fluctuation (RMSF) was calculated based on molecular dynamics simulation trajectories.

According to the I-T-P network, AKT1/C11, MAPK14/C1, and MAPK14/C4 are the possible ligand–receptor complexes for combining AKT1 and MAPK14. The molecular binding was also simulated through molecular dynamics simulation. As shown in Figure 8A1–C1, all systems of the protein reach convergence, indicating that the simulation finally stabilizes.

For AKT1/C11, the protein–ligand complex part converged at 40 ns and was very stable in the subsequent simulations. The RMSD of the C11 simulation was within 2 Å throughout, with only weak fluctuations occurring around 67 ns. For MAPK14/C1, the RMSD of the protein–ligand complex was found to converge at 15 ns and fluctuate around 3 Å in the later part of the simulation, while the RMSD of the C1 remained stable throughout the simulation, with an RMSD within 1 Å. In the case of MAPK14/C4, the RMSD of the complex system was larger and less stable than MAPK14/C1. Nevertheless, the RMSD of the small molecule fluctuated within 2 Å, indicating that the small molecule can still bind stably in the active site. The RMSF characterizes the rigidity of the ligand–receptor complex. Higher rigidity indicates more stable binding and superior enzymatic activity. As shown in Figure 8A2–C2, the RMSF of the whole protein was consistently found to be within 2 Å in the three systems, indicating the high rigidity of protein achieved by ligand binding. The RMSF of MAPK14 was lower when combined with C4 compared with C1, indicating that C4 improved MAPK14 rigidity.

**Figure 9:**
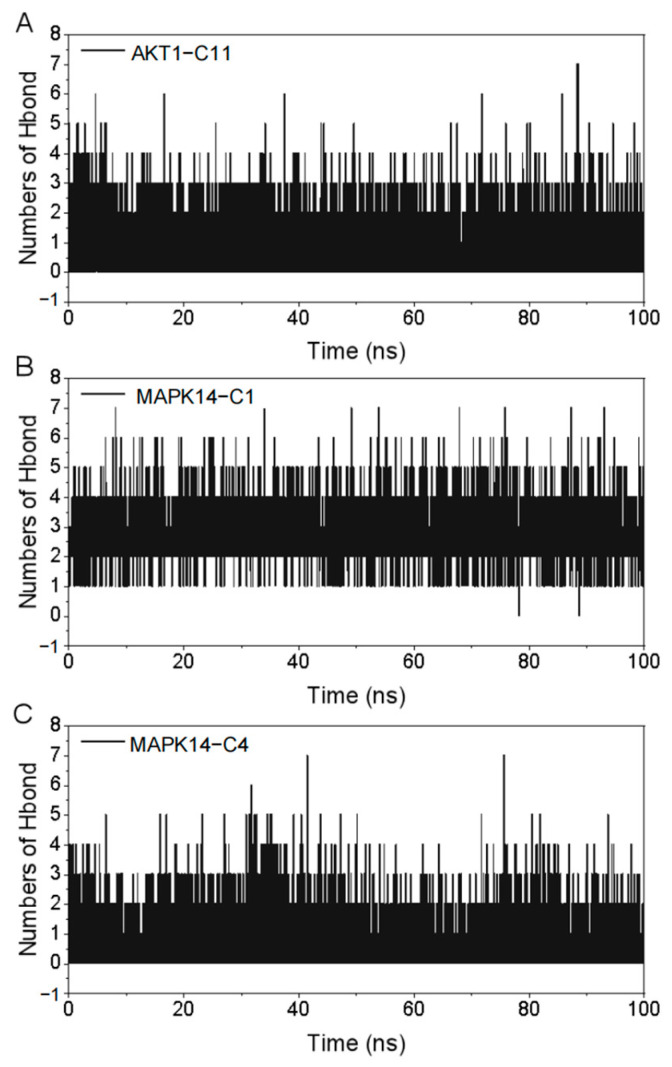
Changes in the number of hydrogen bonds between ligands and proteins ((**A**): AKT1/C11; (**B**): MAPK14/C1; (**C**): MAPK14/C4.) during the molecular dynamics simulation.

Hydrogen bonding is one of the strongest noncovalent interactions; the higher the number of hydrogen bonds, the more favorable the binding of small molecules and proteins. The number of hydrogen bonds formed between ligands and proteins during the simulation was ranked as MAPK14/C1>AKT1/C11>MAPK14/C4. MAPK14/C1 formed the largest number of hydrogen bonds, which may be the reason that MAPK14/C1 had the strongest stability of the abovementioned complexes, as shown in Figure 9.

### 3.7. Effect of PF on Bone Marrow Mesenchymal Stem Cell (BMSC) Viability

**Figure 10:**
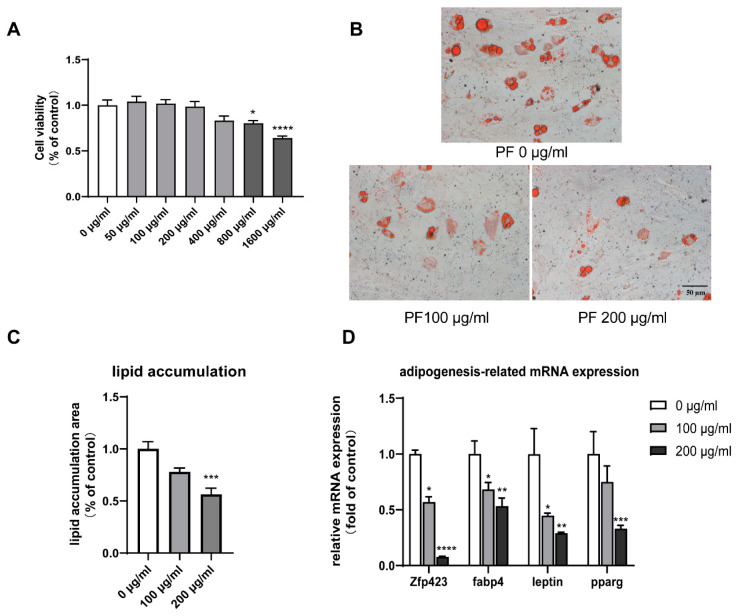
(**A**). Toxicity test for PF extract. PF extract of no more than 400 μg/mL showed no significant inhibition against BMSC proliferation. (**B**). Red Oil staining of adipocytes on Day 10. (**C**). The quantification of lipid accumulation by Red Oil staining (B) using Image J. (**D**). RT-qPCR showed decreased white adipocyte-related gene expression. * *p* < 0.05, ** *p* < 0.01, *** *p* < 0.001, **** *p* < 0.0001.

BMSCs were co-cultured with PF at different concentrations, as shown in Figure 10A. The CCK8 kit was used to test cell viability after eight days of intervention. It revealed that PF with a concentration of no more than 400 μg/mL demonstrated no significant cytotoxicity against the viability of BMSCs.

### 3.8. PF Inhibited Lipid Accumulation and Downregulated Adipogenesis-Related Gene Expression

ORO staining revealed a notable dose-dependent decrease in lipid accumulation, which indicated the inhibition of adipogenesis in PF-treated adipocytes, as shown in Figure 10B,C. BMSCs treated with 200 μg/mL PF showed the downregulated expression of white adipocyte-specific genes Zfp423 and leptin, as well as the pan-adipogenesis genes fabp4 and pparg, as shown in Figure 10D.

### 3.9. PF Promoted Mitochondrial Biogenesis and Upregulated Brite Adipocyte-Related Gene Expression

**Figure 11:**
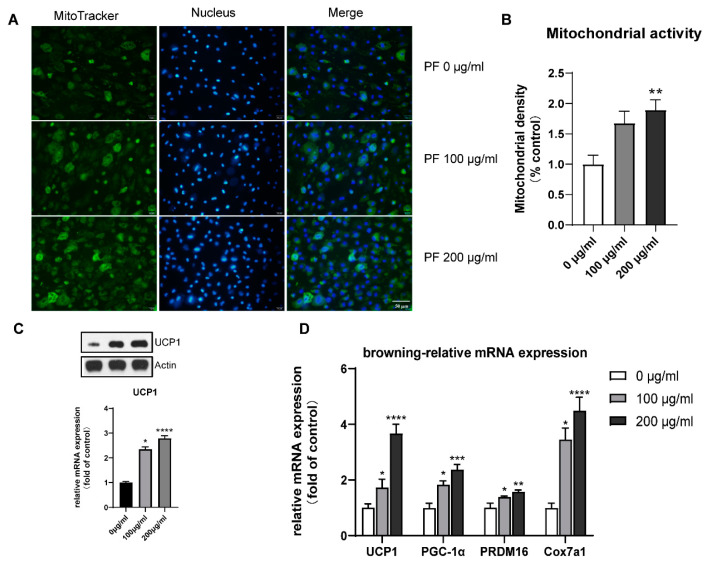
(**A**). Mitochondrial staining of BMSCs with different concentrations of PF extract. (**B**). Quantification of staining (A) using Image J. (**C**). Western blot showed increased UCP1 expression when treated with increased PF extract concentration. (**D**). RT-qPCR showed an upregulated expression of brite-related gene expression. * *p* < 0.05, ** *p* < 0.01, *** *p* < 0.001, **** *p* < 0.0001.

Mito-tracker staining showed increased mitochondrial activity when treated with 200 μg/mL PF, as shown in Figure 11A,B. The RT-qPCR also showed that PF intervention could promote the expression of the brite adipocyte-related genes PGC-1α, PRDM16, and Cox7a1. The results of the Western blot, as well as RT-qPCR, showed the elevated expression of the browning-specific marker UCP1, as illustrated in Figure 11C,D.

### 3.10. Effect of PF on Browning Could Be Mediated by the p38 MAPK Pathway as Well as PI3K-AKT Pathway

**Figure 12:**
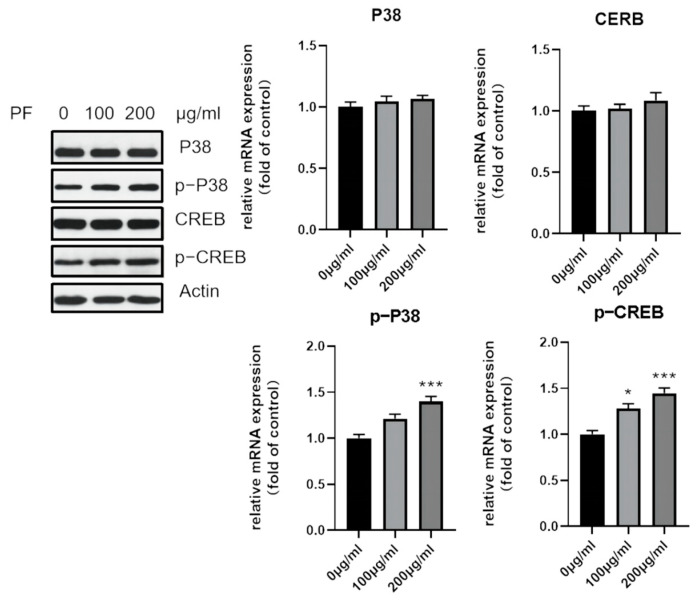
Western blot showed increased phosphorylation of p38, as well as CREB, after PF extract treatment. * *p* < 0.05, *** *p* < 0.001.

**Figure 13:**
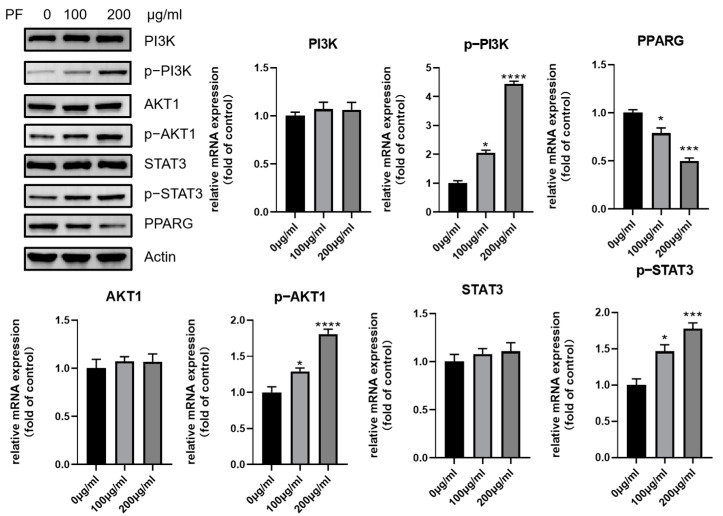
Western blot showed increased phosphorylation of PI3K, AKT1, and STAT3 after PF extract treatment. However, the expression of PPARG was downregulated after PF intervention. * *p* < 0.05, *** *p* < 0.001, **** *p* < 0.0001.

The expression of core genes screened was validated by Western blot, as shown in Figure 12 and Figure 13. It seems that PF intervention did not change the expression level of p38 MPAK (MAPK14), AKT1, and STAT3, but enhanced their phosphorylation level. As for PPARG, the expression was downregulated.

The network pharmacology prediction showed that the p38 MAPK and PI3K-AKT pathways could be the potential pathways involved in adipocyte browning. As a result, the expression of p38 MAPK and its phosphorylation was examined, and an increased expression was observed after treatment with PF. The phosphorylation of downstream transcription factor CREB was also found to have an increased expression, as shown in Figure 12. Similarly, the expression of PI3K and AKT1, as well as their phosphorylation, were also examined. It also showed an increased phosphorylation level after the intervention of PF.

**Figure 14:**
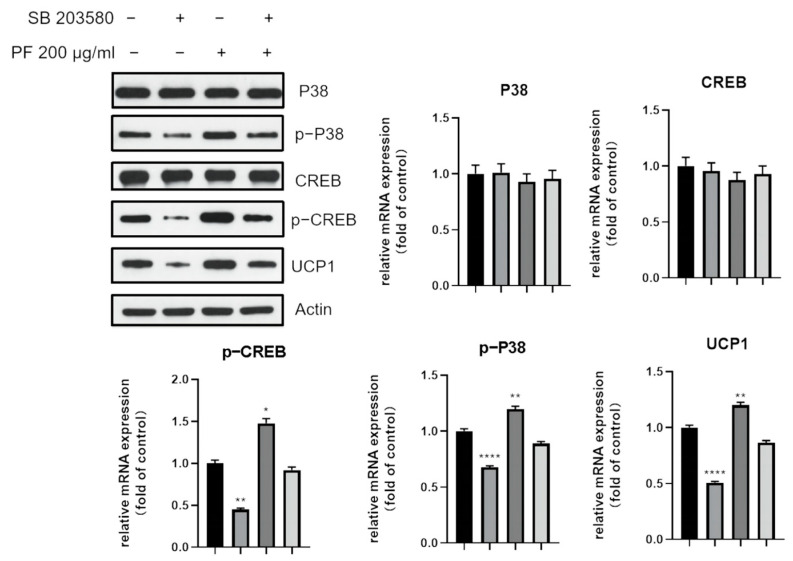
Western blot showed that the increased expression of the thermogenic UCP1, as well as p-P38 and p-CREB, was downregulated after the addition of the p38 MAPK inhibitor SB203580. * *p* < 0.05, ** *p* < 0.01, **** *p* < 0.0001.

To further confirm the role of the p38 MAPK and PI3K-AKT pathway in adipocyte browning, the expression of browning-specific marker UCP1 and the phosphorylation of p38 were examined after intervention with p38 MAPK inhibitor SB203580. As shown in Figure 14, the increased expression of UCP1, as well as p-P38, was downregulated after the addition of the inhibitor.

**Figure 15:**
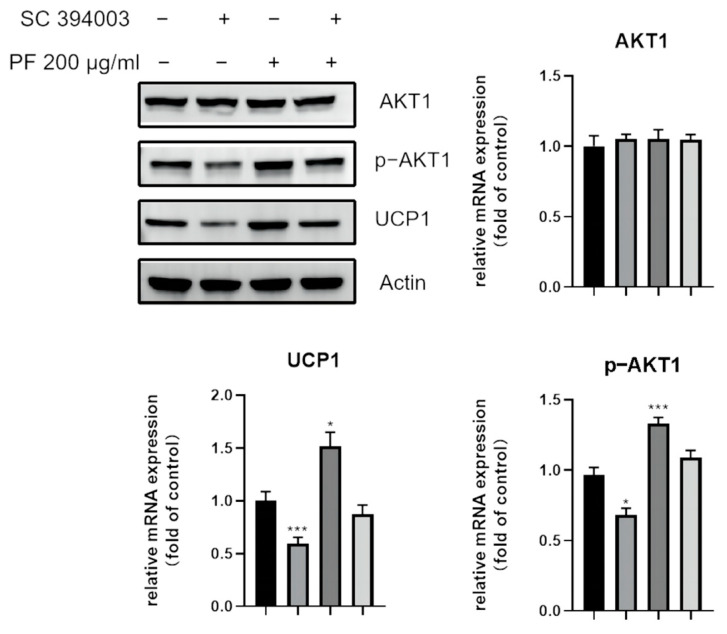
Western blot showed that the increased expression of the thermogenic UCP1, as well as p-AKT1 was downregulated after the addition of the AKT inhibitor SC394003. * *p* < 0.05, *** *p* < 0.001.

Similarly, the UCP1 expression and the phosphorylation of AKT1 was examined after being treated with AKT inhibitor SC394003, which inhibited the increased expression of UCP1 as well p-AKT1, as shown in Figure 15. In all, PF could promote browning via the p38 MAPK as well as PI3K-AKT pathway, leading to the differentiation into brite adipocytes.

## 4. Discussion

Due to changes in diet and genetic factors, the number of obese people is increasing year by year [37]. When energy intake exceeds energy depletion for a long time, excess nutrients are converted into triglycerides and stored in fat cells. This process is accompanied by the proliferation and differentiation of adipose stem cells and the increase in lipid storage, which leads to the proliferation of adipose tissue. Obesity increases the risk of cardiovascular and cerebrovascular diseases, diabetes, cancer, and other noninfectious diseases, seriously endangering human health [38].

Currently, obesity is mainly treated with surgery or drugs to reduce the body’s energy intake. Orlistat is an intestinal fat inhibitor, which can achieve weight loss by reducing the absorption of fat in food, but resulting in a deficiency of fat-soluble vitamins in the long term [39]. Liraglutide is an analog of glucagon-like peptide-1, which increases the risk of hypoglycemia and acute pancreatitis [40]. Qsymia is a type of weight-loss drug aimed at the central nervous system, accompanied by side effects such as headache, sleep loss, constipation, and vertigo [41]. So far, the most-adopted therapeutic strategy is to treat obesity by reducing energy intake. However, it also produces many side effects related to it. As a consequence, increasing energy consumption could become a promising strategy for treating obesity.

White adipocytes can be transformed into brown-like adipocytes. This process is called “Browning” [42]. Currently, it is considered that the browning of white adipocytes is an effective measure against obesity [43]. PF (the dry leaf of the Labiatae plant *P. frutescens*) has various effects, such as regulating glucose and lipid metabolism, antioxidation, antidepression, and relieving cough and asthma [44,45,46]. Studies have reported that the total flavonoid extract of *Perilla* leaves can regulate glucose and lipid metabolism disorders in diabetic model mice and has a good antidiabetic effect [47]. In this study, the browning effect promoted by PF was analyzed via the combination of network pharmacology and in vitro validation to explore a new therapeutical approach against obesity.

The GO enrichment analysis demonstrated that the top enriched biological processes were the intracellular receptor-signaling pathway, activation of protein kinase activity, response to peptide hormone, regulation of lipid metabolic process, and peptidyl-serine phosphorylation, which are responsible for signal transduction. The CC enrichment showed that these proteins were mainly located on the membrane raft, membrane microdomain, vesicle lumen, transferase complex, and transcription regulator complex, indicating that they are mostly located within the cells. The main functions of these potential target genes are related to nuclear receptor activity, ligand-activated transcription factor activity, protein serine/threonine kinase activity, DNA-binding transcription-factor binding, and phosphatase binding, which are mostly related to the signal-transduction cascade. In all, the GO enrichment results indicate that the target genes enriched are mostly responsible for intracellular signal transduction within the cell.

A total of 56 target genes of PF were overlapped and selected as potential targets for the promotion of adipocyte browning. Core targets were screened out by PPI network analysis and the MCODE plugin. It was interesting to note that AKT- and MAPK-related targets (AKT1, AKT2, MAPK14) were shown in cluster 1, indicating that they may play an important role in the PPI network. MAPK14 belongs to the p38 MAPK family, whose function is to initiate signal-transduction cascades that can finally activate transcription factors when cells are simulated by external stimulation, such as stress or proinflammatory cytokines [48].

The KEGG pathway analysis indicated that lipid and atherosclerosis, the PI3K-Akt signaling pathway, the estrogen signaling pathway, and the p38 MAPK signaling pathway might be involved in the browning process, stimulated by PF. P38 MAPKs initiate downstream signal transduction via phosphorylation. It has been reported that there are more than 200 substrates, including some kinases, leading to phosphorylation cascades, such as CREB1, ATF1, STAT1, and STAT3 [49]. The p38 MAPK pathway has been widely studied. Lee et al. reported that adapalene, an anti-acne agent with retinoic acid receptor agonism, induces adipose browning through the RARβ-p38 MAPK-ATF2 pathway [50]. Mukherjee et al. found that the addition of prednisone to 3T3-L1 pre-adipocytes can promote browning, characterized by the upregulation of browning-specific genes, such as UCP1, PGC-1α, and PRDM16 [51]. The activation is mediated by the p38 MAPK pathway, activating the transcriptional factor ATF2. Wu et al. found a mesencephalic astrocyte-derived neurotrophic factor (Manf), which is a feeding-induced hepatokine that can ameliorate diet-induced obesity by promoting adipose browning via the p38 MAPK pathway [52].

Several studies have also shown that the PI3K-Akt signaling pathway can play a part during the browning process. Zhao et al. showed that Vitamin D3 could inactivate the PI3K/Akt/mTOR/p53 signaling pathway and inhibit the expression of browning-specific markers but could promote autophagy [53]. Another study showed that the downregulation of osteopontin demonstrated a negative effect on the browning of WAT and downregulated the expression of PPARγ via the PI3K-AKT pathway [54]. Molecular docking and molecular dynamic simulation confirmed the stability of ligand binding with MAPK14 and AKT1.

The in vitro experiments were conducted to validate the browning effect of PF based on the BMSCs. The browning process is accompanied by an increase in browning markers, including uncoupling protein-1 (UCP-1), pparγ-assisted activator 1α (PGC-1α), and positive regulatory domain containing 16 (PRDM16) [55]. The results of the Western blot, as well as RT-qPCR, showed the elevated expression of the browning-specific marker UCP1, the typical brite adipocyte-specific marker. Mito-tracker staining showed increased mitochondrial activity when treated with 200 μg/mL PF, and RT-qPCR showed that PF intervention could promote the expression of thermogenic genes PGC-1α, PRDM16, and Cox7a1. The phosphorylation of p38 MAPK, as well as AKT1, was examined, and an increased expression was both observed after treatment with PF. The role of the p38 MAPK pathway as well as the PI3K-AKT pathway in adipocyte browning was further confirmed by the p38 MAPK inhibitor SB203580 and AKT inhibitor SC394003. In all, the in vitro validation confirmed that PF could promote adipocyte browning via the p38 MAPK as well as the PI3K-ATK pathway, leading to the differentiation of brite adipocytes.

As for the limitation of the present study, only a small part of the mechanisms or pathways by which PF promotes adipocyte browning were analyzed and discussed. More browning- or adipogenesis-related genes can be examined by RT-qPCR to explore more possible mechanisms. Further studies are needed to validate the potential role of more pathways, such as estrogen signal pathways.

## 5. Conclusions

In conclusion, the study revealed the therapeutic function of PF and possible mechanisms involved in the promotion of adipocyte browning. It was demonstrated that PF can promote adipocyte browning by the P38 MAPK pathway and the PI3K-AKT pathway. The study showed great potential in combing in silicon methods with experimental validation to investigate the biological function of herb extracts.

## Figures and Tables

**Table 1:** Information of 17 bioactive compounds from PF that predicted the 56 overlapped genes.

Compound	PubChem CID	Label
1-(2,4,5-Triethoxyphenyl)propan-2-amine	44719557	C1
19435-97-3	3084311	C2
Cumic Acid	10820	C3
Dibutyl phthalate	3026	C4
Diisobutyl phthalate	6782	C5
Eugenol	3314	C6
Isoeugenol	853433	C7
Luteolin	5280445	C8
Methyl Caffeate	689075	C9
Methyl Protocatechuate	287064	C10
Nerolidyl Acetate	5363426	C11
Nonanoic Acid	8158	C12
Patchouli Alcohol	146159297	C13
Safrol	5144	C14
Tau-Cadinol	160799	C15
Vanillic Acid	8468	C16
Zoomaric Acid	445638	C17

## Data Availability

The datasets generated during and/or analyzed during the current study are available from the corresponding author on reasonable request.

## Supplementary Materials

The following supporting information can be downloaded at: https://www.mdpi.com/article/10.3390/nu15061487/s1, Table S1: Primer sequences used for semi-quantitative RT-PCR analysis.

## References

[1] Nakanishi H., Matar R.H., Vahibe A., Abu Dayyeh B.K., Galvani C., Pullatt R., Davis S.S., Clapp B., Ghanem O.M. (2022). Single Versus Double Anastomosis Duodenal Switch in the Management of Obesity: A Meta-analysis and Systematic Review. Surg. Laparosc. Endosc. Percutan. Tech..

[2] Dickerson R.N., Andromalos L., Brown J.C., Correia M., Pritts W., Ridley E.J., Robinson K.N., Rosenthal M.D., van Zanten A.R.H. (2022). Obesity and critical care nutrition: Current practice gaps and directions for future research. Crit. Care.

[3] Klein Hazebroek M., Keipert S. (2022). Obesity-resistance of UCP1-deficient mice associates with sustained FGF21 sensitivity in inguinal adipose tissue. Front. Endocrinol..

[4] Sakaguchi M., Okagawa S., Okubo Y., Otsuka Y., Fukuda K., Igata M., Kondo T., Sato Y., Yoshizawa T., Fukuda T. (2022). Phosphatase protector alpha4 (alpha4) is involved in adipocyte maintenance and mitochondrial homeostasis through regulation of insulin signaling. Nat. Commun..

[5] Lee D.K., Kim T., Byeon J., Park M., Kim S., Kim J., Choi S., Lee G., Park C., Lee K.W. (2022). REDD1 promotes obesity-induced metabolic dysfunction via atypical NF-kappaB activation. Nat. Commun..

[6] Herzig S., Shaw R.J. (2018). AMPK: Guardian of metabolism and mitochondrial homeostasis. Nat. Rev. Mol. Cell Biol..

[7] Kwan H.Y., Wu J., Su T., Chao X.J., Liu B., Fu X., Chan C.L., Lau R.H.Y., Tse A.K.W., Han Q.B. (2017). Cinnamon induces browning in subcutaneous adipocytes. Sci. Rep..

[8] Wang J., Zhang L., Dong L., Hu X., Feng F., Chen F. (2019). 6-Gingerol, a Functional Polyphenol of Ginger, Promotes Browning through an AMPK-Dependent Pathway in 3T3-L1 Adipocytes. J. Agric. Food Chem..

[9] Thomas S.S., Kim M., Lee S.J., Cha Y.S. (2018). Antiobesity Effects of Purple Perilla (*Perilla frutescens* var. *acuta*) on Adipocyte Differentiation and Mice Fed a High-fat Diet. J. Food Sci..

[10] Zheng F., Quan H.-Y., Quan J., Yin X. (2017). Effect of Perillae Folium Extract on 3T3-L1 Adipocytes. Lishizhen Med. Mater. Med. Res..

[11] Ying P., Fei H.Y., Quan H.Y. (2017). Effects and mechanism research on Perillae folium extract in obese mice. Zhong Hua Zhong Yi Yao Za Zhi.

[12] Cheng Y., Liu H., Li J., Ma Y., Song C., Wang Y., Li P., Chen Y., Zhang Z. (2022). Monascin abrogates RANKL-mediated osteoclastogenesis in RAW264.7 cells via regulating MAPKs signaling pathways. Front. Pharmacol..

[13] He Q., Yang J., Chen D., Li Y., Gong D., Ge H., Wang Z., Wang H., Chen P. (2022). 12-Deoxyphorbol-13-Hexadecanoate Abrogates OVX-Induced Bone Loss in Mice and Osteoclastogenesis via Inhibiting ROS Level and Regulating RANKL-Mediated NFATc1 Activation. Front. Pharm..

[14] Liu Y., Zhong H., Xu P., Zhou A., Ding L., Qiu J., Wu H., Dai M. (2022). Deciphering the combination mechanisms of Gualou-Xiebai herb pair against atherosclerosis by network pharmacology and HPLC-Q-TOF-MS technology. Front. Pharmacol..

[15] Wang Y.X., Yang Z., Wang W.X., Huang Y.X., Zhang Q., Li J.J., Tang Y.P., Yue S.J. (2022). Methodology of network pharmacology for research on Chinese herbal medicine against COVID-19: A review. J. Integr. Med..

[16] Zhang G.B., Li Q.Y., Chen Q.L., Su S.B. (2013). Network pharmacology: A new approach for chinese herbal medicine research. Evid. Based Complement. Alternat. Med..

[17] Wu Q., Li X., Jiang X.W., Yao D., Zhou L.J., Xu Z.H., Wang N., Zhao Q.C., Zhang Z. (2022). Yuan-Zhi decoction in the treatment of Alzheimer’s disease: An integrated approach based on chemical profiling, network pharmacology, molecular docking and experimental evaluation. Front. Pharmacol..

[18] Sharma B., Bhattacherjee D., Zyryanov G.V., Purohit R. (2022). An insight from computational approach to explore novel, high-affinity phosphodiesterase 10A inhibitors for neurological disorders. J. Biomol. Struct. Dyn..

[19] Bhardwaj V.K., Purohit R. (2022). A lesson for the maestro of the replication fork: Targeting the protein-binding interface of proliferating cell nuclear antigen for anticancer therapy. J. Cell Biochem..

[20] Singh R., Bhardwaj V.K., Purohit R. (2022). Computational targeting of allosteric site of MEK1 by quinoline-based molecules. Cell Biochem. Funct..

[21] Que W., Wu Z., Chen M., Zhang B., You C., Lin H., Zhao Z., Liu M., Qiu H., Cheng Y. (2021). Molecular Mechanism of Gelsemium elegans (Gardner and Champ.) Benth. Against Neuropathic Pain Based on Network Pharmacology and Experimental Evidence. Front. Pharmacol..

[22] An J., Fan H., Han M., Peng C., Xie J., Peng F. (2022). Exploring the mechanisms of neurotoxicity caused by fuzi using network pharmacology and molecular docking. Front. Pharmacol..

[23] Trott O., Olson A.J. (2010). AutoDock Vina: Improving the speed and accuracy of docking with a new scoring function, efficient optimization, and multithreading. J. Comput. Chem..

[24] Ling J., Huang Y., Sun Z., Guo X., Chang A., Pan J., Zhuo X. (2022). Exploration of the effect of Celastrol on protein targets in nasopharyngeal carcinoma: Network pharmacology, molecular docking and experimental evaluations. Front. Pharmacol..

[25] Liao Y., Ding Y., Yu L., Xiang C., Yang M. (2022). Exploring the mechanism of Alisma orientale for the treatment of pregnancy induced hypertension and potential hepato-nephrotoxicity by using network pharmacology, network toxicology, molecular docking and molecular dynamics simulation. Front. Pharmacol..

[26] Nakano M., Champagne B. (2016). Nonlinear optical properties in open-shell molecular systems. Wiley Interdiscip. Rev. Comput. Mol. Sci..

[27] Mottola D.M., Laiter S., Watts V.J., Tropsha A., Wyrick S.D., Nichols D.E., Mailman R.B. (1996). Conformational analysis of D1 dopamine receptor agonists: Pharmacophore assessment and receptor mapping. J. Med. Chem..

[28] Maier J.A., Martinez C., Kasavajhala K., Wickstrom L., Hauser K.E., Simmerling C. (2015). ff14SB: Improving the Accuracy of Protein Side Chain and Backbone Parameters from ff99SB. J. Chem. Theory Comput..

[29] Yang H., Sun W., Ma P., Yao C., Fan Y., Li S., Yuan J., Zhang Z., Li X., Lin M. (2020). Multiple Components Rapidly Screened from Perilla Leaves Attenuate Asthma Airway Inflammation by Synergistic Targeting on Syk. J. Inflamm. Res..

[30] Bae J.S., Han M., Shin H.S., Kim M.K., Shin C.Y., Lee D.H., Chung J.H. (2017). Perilla frutescens leaves extract ameliorates ultraviolet radiation-induced extracellular matrix damage in human dermal fibroblasts and hairless mice skin. J. Ethnopharmacol..

[31] Zhao Y., Kong H., Zhang X., Hu X., Wang M. (2019). The effect of Perilla (*Perilla frutescens*) leaf extracts on the quality of surimi fish balls. Food Sci. Nutr..

[32] Lin Z., He H., Wang M., Liang J. (2019). MicroRNA-130a controls bone marrow mesenchymal stem cell differentiation towards the osteoblastic and adipogenic fate. Cell Prolif..

[33] Li H., Xie H., Liu W., Hu R., Huang B., Tan Y.F., Xu K., Sheng Z.F., Zhou H.D., Wu X.P. (2009). A novel microRNA targeting HDAC5 regulates osteoblast differentiation in mice and contributes to primary osteoporosis in humans. J. Clin. Investig..

[34] Chen C.C., Kuo C.H., Leu Y.L., Wang S.H. (2021). Corylin reduces obesity and insulin resistance and promotes adipose tissue browning through SIRT-1 and beta3-AR activation. Pharmacol. Res..

[35] Jung T.W., Hwang E.J., Pyun D.H., Kim T.J., Lee H.J., Abd El-Aty A.M., Bang J.S., Kim H.C., Jeong J.H. (2021). 3-hydroxymorphinan enhances mitochondrial biogenesis and adipocyte browning through AMPK-dependent pathway. Biochem. Biophys. Res. Commun..

[36] Hsin K.Y., Ghosh S., Kitano H. (2013). Combining machine learning systems and multiple docking simulation packages to improve docking prediction reliability for network pharmacology. PLoS ONE.

[37] Kolotkin R.L., Meter K., Williams G.R. (2001). Quality of life and obesity. Obes. Rev..

[38] Vecchie A., Dallegri F., Carbone F., Bonaventura A., Liberale L., Portincasa P., Fruhbeck G., Montecucco F. (2018). Obesity phenotypes and their paradoxical association with cardiovascular diseases. Eur. J. Intern. Med..

[39] Filippatos T.D., Derdemezis C.S., Gazi I.F., Nakou E.S., Mikhailidis D.P., Elisaf M.S. (2008). Orlistat-associated adverse effects and drug interactions: A critical review. Drug Saf..

[40] Kelly A.S., Auerbach P., Barrientos-Perez M., Gies I., Hale P.M., Marcus C., Mastrandrea L.D., Prabhu N., Arslanian S., Investigators N.N.T. (2020). A Randomized, Controlled Trial of Liraglutide for Adolescents with Obesity. N. Engl. J. Med..

[41] Cameron F., Whiteside G., McKeage K. (2012). Phentermine and topiramate extended release (Qsymia): First global approval. Drugs.

[42] Ghaben A.L., Scherer P.E. (2019). Adipogenesis and metabolic health. Nat. Rev. Mol. Cell Biol..

[43] Li G., Xie C., Lu S., Nichols R.G., Tian Y., Li L., Patel D., Ma Y., Brocker C.N., Yan T. (2017). Intermittent Fasting Promotes White Adipose Browning and Decreases Obesity by Shaping the Gut Microbiota. Cell Metab..

[44] Li J., Zhang Q., Zhuo Y., Fang Z., Che L., Xu S., Feng B., Lin Y., Jiang X., Zhao X. (2022). Effects of Multi-Strain Probiotics and Perilla frutescens Seed Extract Supplementation Alone or Combined on Growth Performance, Antioxidant Indices, and Intestinal Health of Weaned Piglets. Animals.

[45] Maeda A., Fujimura T., Hirakawa N., Baba K., Kawamoto S. (2022). A Methoxyflavanone from Perilla frutescens Induces Cellular Senescence in A549 Human Lung Adenocarcinoma Cells but Not in Normal Human Bronchial Epithelial Cells. Biol. Pharm. Bull..

[46] Wang Z., Jin X., Zhang X., Xie X., Tu Z., He X. (2022). From Function to Metabolome: Metabolomic Analysis Reveals the Effect of Probiotic Fermentation on the Chemical Compositions and Biological Activities of Perilla frutescens Leaves. Front. Nutr..

[47] Wang Z.X., Lin Q.Q., Tu Z.C., Zhang L. (2020). The influence of in vitro gastrointestinal digestion on the Perilla frutescens leaf extract: Changes in the active compounds and bioactivities. J. Food Biochem..

[48] Xia T., Ma J., Sun Y., Sun Y. (2022). Androgen receptor suppresses inflammatory response of airway epithelial cells in allergic asthma through MAPK1 and MAPK14. Hum. Exp. Toxicol..

[49] Ding Q.Y., Zhang Y., Ma L., Chen Y.G., Wu J.H., Zhang H.F., Wang X. (2020). Inhibiting MAPK14 showed anti-prolactinoma effect. BMC Endocr. Disord..

[50] Lee N.H., Choi M.J., Yu H., Kim J.I., Cheon H.G. (2022). Adapalene induces adipose browning through the RARbeta-p38 MAPK-ATF2 pathway. Arch. Pharm. Res..

[51] Mukherjee S., Yun J.W. (2022). Prednisone stimulates white adipocyte browning via beta3-AR/p38 MAPK/ERK signaling pathway. Life Sci..

[52] Wu T., Liu Q., Li Y., Li H., Chen L., Yang X., Tang Q., Pu S., Kuang J., Li R. (2021). Feeding-induced hepatokine, Manf, ameliorates diet-induced obesity by promoting adipose browning via p38 MAPK pathway. J. Exp. Med..

[53] Zhao Y., Qin R. (2022). Vitamin D3 affects browning of white adipocytes by regulating autophagy via PI3K/Akt/mTOR/p53 signaling in vitro and in vivo. Apoptosis.

[54] Lu Y., Xu Y., Yuan W., Wang M., Zhou Y., Chen K., Huang Q. (2020). Downregulation of osteopontin inhibits browning of white adipose tissues through PI3K-AKT pathway in C57BL/6 mice. Eur. J. Pharmacol..

[55] Bargut T.C.L., Souza-Mello V., Aguila M.B., Mandarim-de-Lacerda C.A. (2017). Browning of white adipose tissue: Lessons from experimental models. Horm. Mol. Biol. Clin. Investig..

