# Supplementary material for: Leaf Extract of Perilla frutescens (L.) Britt Promotes Adipocyte Browning via the p38 MAPK Pathway and PI3K-AKT Pathway
Source: Nutrients. 2023 Mar 20;15(6):1487. doi: 10.3390/nu15061487 (PMC10054491; doi:10.3390/nu15061487)
Supplement: Supplementary file 1 [file nutrients-15-01487-s001.zip › Supplemental Table S1.pdf]

Table S1 Primer sequences used for semi-quantitative RT-PCR analysis

gene	Forward primer	Reserve primer
β-actin	AACAGTCCGCCTAGAAGCAC	CGTTGACATCCGTAAAGACC
Ucp1	TCTCAGCCGGCTTAATGACTG	GGCTTGCATTCTGACCTTCAC
Pgc-1α	GAAAGGGCCAAACAGAGAGA	GTAAATCACACGGCGCTCTT
Prdm16	ACACGCCAGTTCTCCAACCTGT	TGCTTGTTGAGGGAGGAGGTA
Cidea	TCCTATGCTGCACAGATGACG	TGCTCTTCTGTATCGCCCAGT
Zfp423	CAGGCCCACAAGAAGAACAAG	GTATCCTCGCAGTAGTCGCACA
Fabp4	GATGAAATCACCGCAGACGAC	ATTCCACCACCAGCTTGTCAC
Leptin	TGAGTTTGTCCAAGATGGACC	GCCATCCAGGCTCTCTGG
Pparg	TGCTGTTATGGGTGAAACTCTG	CTGTGTCAACCATGGTAATTTCTT
Cox7a1	CAGCGTCATGGTCAGTCTGT	AGAAAACCGTGTGGCAGAGA
